# The Novel Notch-induced Long Noncoding RNA LUNAR1 Determines the Proliferation and Prognosis of Colorectal Cancer

**DOI:** 10.1038/s41598-019-56536-2

**Published:** 2019-12-27

**Authors:** Zixi Zhang, Gai Li, He Qiu, Jingyi Yang, Xin Bu, Shaojun Zhu, Jianyong Zheng, Chengxue Dang, Weizhong Wang, Dake Chu

**Affiliations:** 1 Department of Gastroenterology, the First Affiliated Hospital of Xi’an Jiaotong University, Xi’an, China; 2 Department of Dermatology, the First Affiliated Hospital of Xi’an Jiaotong University, Xi’an, China; 3 Information Center, the First Affiliated Hospital of Xi’an Jiaotong University, Xi’an, China; 4 State Key Laboratory of Cancer Biology and Department of Biochemistry and Molecular Biology, Fourth Military Medical University, Xi’an, Shaanxi China; 5 Department of Pathology, Tangdu Hospital, the Fourth Military Medical University, Xi’an, Shaanxi China; 6 Department of Gastrointestinal Surgery, Xijing Hospital of Digestive Diseases, the Fourth Military Medical University, Xi’an, China; 7 Department of Surgical Oncology, the First Affiliated Hospital of Xi’an Jiaotong University, Xi’an, China

**Keywords:** Colorectal cancer, Oncogenes, Tumour biomarkers

## Abstract

In contrast to what is known about the complicated roles of Notch signalling in human malignancies, the direct target genes of Notch signalling are still unclear. Recently, long noncoding RNAs (lncRNAs) have been found to play various roles in the post-transcriptional regulation of gene expression. In the present study, we investigated the potential role of the Notch-induced lncRNA LUNAR1 in colorectal cancer (CRC). We recruited 196 cases of clinical CRC specimens and investigated LUNAR1 levels in these specimens. The associations of LUNAR1 with tumour aggressiveness and clinical outcomes were evaluated. Moreover, the impact of LUNAR1 on the malignant behaviour of tumour cells was tested in cell lines. Significantly increased expression of LUNAR1 in clinical CRC specimens was detected compared with that in matching normal tissues. LUNAR1 expression in CRC was found to be associated with the tumour aggressiveness, disease-free survival and overall survival of patients. The downregulation of LUNAR1 in SW620 cells inhibited cell proliferation, migration, invasion and tumour growth while inducing apoptosis. Moreover, the inhibition of LUNAR1 can significantly suppress IGF1 signalling in CRC. These results indicated that LUNAR1 was increased in CRC and might promote tumour progression. Thus, LUNAR1 may constitute a promising prognostic marker for the clinical management of CRC.

## Introduction

The Notch signalling pathway is an evolutionarily conserved pathway that plays an essential role in the regulation of cell fate in most cells types^1^. To date, four receptors of Notch signalling have been discovered in mammals, including Notch1, Notch 2, Notch 3, and Notch 4^2^. Notch signalling can either negatively or positively affect cellular processes, including proliferation, differentiation and apoptosis, depending on the cell type^3^. Previous investigations also revealed an interesting role of Notch signalling in human malignant tumours. Notch signalling acts as a tumour suppressor in some malignancies while acting as an oncogene in other neoplasms^4,5^. In the gastrointestinal tract, Notch signalling has been proven to be essential in cell proliferation and carcinogenesis^6,7^. In addition, our previous studies demonstrated that Notch1 could act as an oncogene in colorectal cancer (CRC)^8^. The expression of the Notch1 intracellular domain in CRC was significantly associated with the overall survival of patients, in which patients with increased levels of Notch1 were more likely to have an unfavourable overall survival^9^. Conversely, Notch2 was identified to play a tumour suppressor role in CRC^10,11^. In contrast to what is known about the complex roles of Notch signalling, only HES and HEY have been confirmed to be direct target genes of Notch signalling. Therefore, the non-canonical pathway might play critical roles in Notch signalling.

Recently, long noncoding RNAs (lncRNAs) have been found to play an essential role in various molecular pathways, including Notch signalling. Specifically, a Notch-regulated lncRNA, LUNAR1, was demonstrated to be a key downstream target gene in human T cell acute lymphoblastic leukaemia (T-ALL). LUNAR1 was found to be a 491-nucleotide transcript located at 15q26.3, with 4 exons and a poly (A) tail. This lncRNA was suggested to be required for efficient T-ALL growth *in vitro* and *in vivo*^12^. However, the molecular regulation in solid tumours is quite different from that in leukaemia. Therefore, the role of the lncRNA LUNAR1 in solid tumours remains to be determined to clarify the contradiction between the complex roles and limited target genes of Notch signalling.

In the present study, we investigated the potential function and subsequent mechanism of the Notch-regulated lncRNA LUNAR1 in human CRC to gain an understanding of lncRNA-mediated non-canonical Notch signalling in human malignancy.

## Results

### Detection of LUNAR1 expression in the study cohort

**Figure 1:**
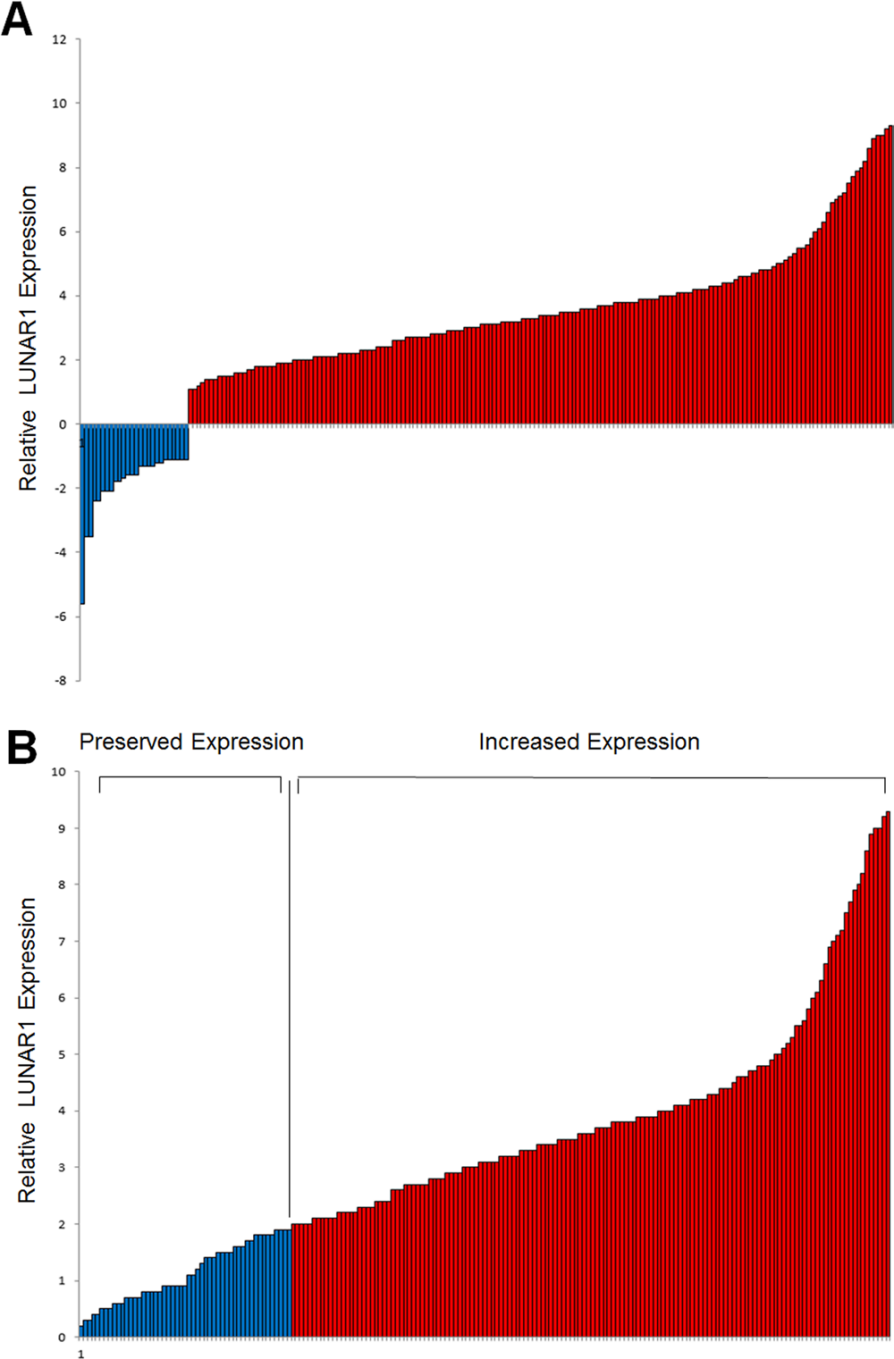
Detection of LUNAR1 expression in clinical CRC specimens. (**A**) Increased LUNAR1 expression was detected in 170 CRC specimens compared with adjacent normal tissues; (**B**) Patients were assigned into two groups according to LUNAR1 level: patients with increased LUNAR1 level (fold change ≥ 2) and preserved LUNAR1 level (fold change < 2).

The characteristics of the 196 patients involved in the study are shown in Table 1. After normalization to the expression of 18S rRNA, LUNAR1 expression in CRC was significantly increased in 86.7% (170/196) of clinical CRC specimens compared with that in adjacent normal tissues (Fig. 1A). This increased expression pattern indicated that aberrant LUNAR1 levels might be associated with CRC carcinogenesis. To facilitate further statistical analysis, we manually assigned patients into two groups: patients with increased LUNAR1 levels (n = 145, fold change ≥ 2) and preserved LUNAR1 levels (n = 51, fold change < 2) (Fig. 1B).

**Table 1:** Statistical results of LUNAR1 expression.

Variable	n	LUNAR1 expression		*P*
		Increased	Preserved	
Total	196	145	51	
Sex				0.151*
Male	124	96	28	
Female	72	49	23	
Age at diagnosis				0.312*
≤60	145	110	35	
>60	51	35	16	
BMI				
Normal weight	130	89	41	0.030*
Overweight	42	34	8	
Obese	24	22	2	
Tumour location				0.090*
Right	66	45	21	
Left	71	59	12	
Rectum	59	41	18	
Tumour size				0.054*
≤3.0 cm	46	29	17	
>3.0 cm	150	116	34	
Differentiation status				0.038*
Well	42	25	17	
Moderate	89	67	22	
Poor	65	53	12	
Depth of invasion				0.013*
T_1_ + T_2_	68	43	25	
T_3_ + T_4_	128	102	26	
Lymph node metastasis				0.024*
Absent (N0)	85	56	29	
Present (N1–3)	111	89	22	
Distant metastasis				0.161^†^
Absent (M0)	174	126	48	
Present (M1)	22	19	3	
TNM stage				0.033^†^
I	31	17	14	
II	54	39	15	
III	89	70	19	
IV	22	19	3	
MSI				0.426*
MSS	168	126	42	
MSI-H	28	19	9	
KRAS mutation				0.315*
(−)	131	94	37	
(+)	65	51	14	
BRAF mutation				0192*
(−)	165	125	40	
(+)	31	20	11	
PIK3CA mutation				0.264*
(−)	160	129	31	
(+)	36	26	10	

*P value when expression levels were compared using Pearson *χ*^2^ test.

^†^*P* value when expression levels were compared using Fisher’s exact test.

### LUNAR1 expression in CRC was correlated with tumour aggressiveness

According to our classification of LUNAR1 expression, we analysed the association of LUNAR1 expression in CRC with clinicopathological factors. Statistical analysis revealed that the difference in LUNAR1 levels among the normal weight, overweight and obesity groups was significant (*P* = 0.030). Subgroup analysis showed that increased LUNAR1 expression was more likely to be detected in obese patients compared with normal weight patients (*P* = 0.020). However, this difference did not reach a statistically significant level between overweight and normal weight patients (*P* = 0.120) or between obese and overweight patients (*P* = 0.246). This association suggested that LUNAR1 might be involved in obesity-related tumourigenesis and progression in CRC. In addition, the expression of LUNAR1 in CRC was also showed to be associated with the tumour differentiation status, invasion depth, node metastasis status and TNM stage, indicating a significant role of LUNAR1 in CRC aggressiveness. No statistically significant association of LUNAR1 with tumour size (*P* = 0.054) or distant metastases (*P* = 0.161) was found. In addition, the associations of LUNAR1 with sex, age, tumour location, MSI, KRAS mutation, BRAF mutation, and PIK3CA mutation were not statistically significant.

### LUNAR1 expression in CRC was correlated with the disease-free survival of patients

**Figure 2:**
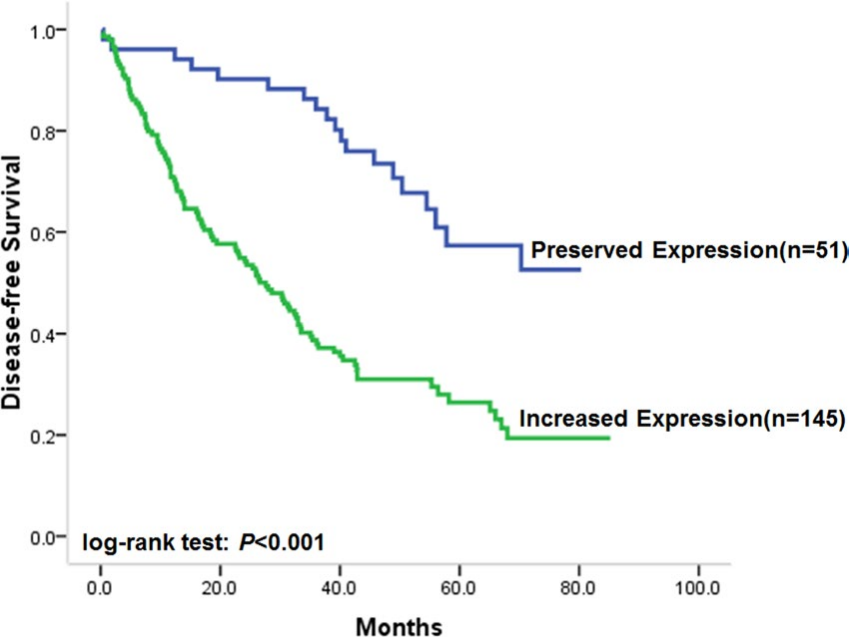
Correlation of LUNAR1 expression with disease-free survival. Kaplan-Meier univariate survival analysis showed that preserved LUNAR1 expression in CRC was significantly associated with favorable DFS of patients.

In the present study cohort, the postoperative median follow-up time of all recruited patients with CRC was 40 months. The association of the LUNAR1 expression level with disease-free survival (DFS) was evaluated by Kaplan-Meier analysis. The univariate analysis results indicated that preserved LUNAR1 expression in CRC was significantly associated with a favourable DFS in patients (Fig. 2, log-rank test: *P* < 0.001). The median DFS of all the recruited patients was 36.4 months (95% CI: 29.6–43.2). According to the subgroup classification by LUNAR1 expression level, the DFS of patients with increased LUNAR1 levels was 26.6 months (95% CI: 20.3.3–32.9); however, DFS could not be estimated in the preserved LUNAR1 expression group due to favourable outcomes. These results indicated that CRC patients with increased LUNAR1 levels were more likely to have tumour recurrence and metastasis. In the Kaplan-Meier analysis, unfavourable DFS was also found to be associated with poor differentiation status (log-rank test: *P* < 0.001), obesity (log-rank test: *P* = 0.005) and TNM stage (log-rank test: *P* < 0.001). The unadjusted HRs of the clinicopathological factors for DFS are shown in Table 2.

**Table 2:** Association of LUNAR1 and clinical factors with disease-free survival in patients with CRC.

	Unadjusted HR* (95% CI)	*P*	Adjusted HR^†^ (95% CI)	*P*
LUNAR1 level	3.25 (1.98–5.31)	<0.001	2.81 (1.69–4.67)	<0.001
Sex	0.92 (0.697–1.40)	0.983	0.91 (0.63–1.32)	0.630
Age	1.24 (0.87–1.77)	0.238	1.03 (0.71–1.49)	0.894
Tumour location	1.11 (0.53–2.32)	0.790	1.18 (0.79–1.77)	0.406
Tumour size	1.82 (0.56–5.95)	0.319	1.50 (0.48–4.67)	0.489
Differentiation status	2.44 (1.45–4.11)	0.001	1.19 (0.36–3.95)	0.780
TNM stage	3.01 (1.60–5.66)	0.001	3.26 (1.71–6.20)	<0.001

*Hazard ratios in univariate models.

^†^Hazard ratios in multivariable models.

Abbreviations: HR, hazard ratio; 95% CI, 95% confidence interval.

Next, multivariate Cox proportional hazards model analysis adjusted for confounding factors, including sex, age, differentiation status, node status, and TNM stage, was utilized to verify the independent prognostic value of LUNAR1 in patients with CRC. The results of multivariate analysis revealed that preserved LUNAR1 expression in CRC was independently associated with favourable DFS in patients after controlling for known prognostic factors. The adjusted HR for patients with tumours of increased LUNAR1 expression was 2.81 (95% CI: 1.69–4.67, *P* < 0.001) compared with that of the LUNAR1 preserved group as a reference. The adjusted HRs of the clinicopathological factors for DFS are shown in Table 2.

### LUNAR1 expression in CRC was correlated with the overall survival of patients

**Figure 3:**
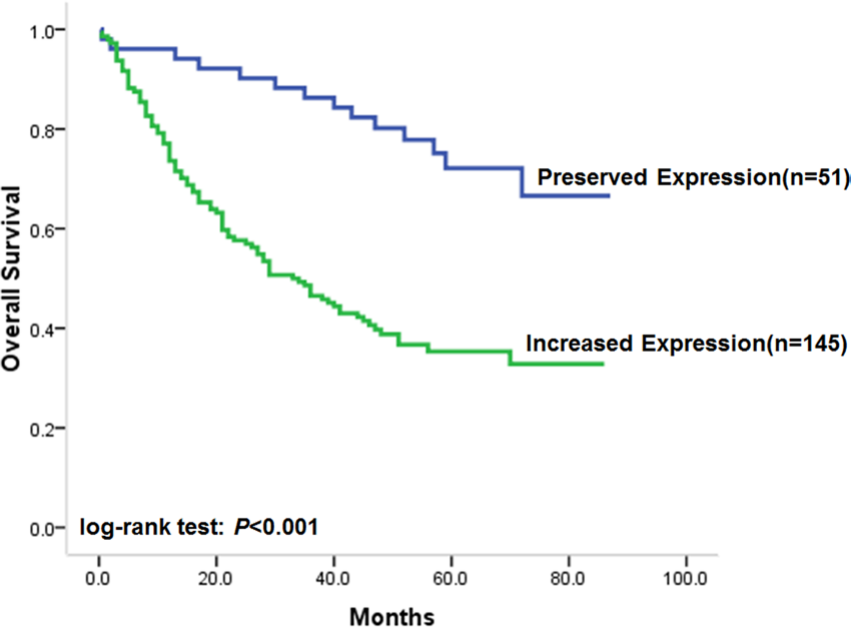
Correlation of LUNAR1 expression with overall survival. Kaplan-Meier univariate survival analysis showed that preserved LUNAR1 expression in CRC was significantly associated with favorable OS of patients.

Considering the association of LUNAR1 with DFS in CRC patients, we next evaluated its correlation with overall survival (OS). As a result, preserved LUNAR1 levels in CRC were significantly associated with favourable OS, similar to its association with DFS (Fig. 3, log-rank test: *P* < 0.001). The OS time of patients with increased LUNAR1 levels was 33.0 months (95% CI: 23.3–42.7), but it could not be estimated in patients with preserved LUNAR1 expression because over half of the patients survived 5 years. In the univariate analysis of clinicopathological factors, unfavourable OS was found to be associated with poor differentiation status (log-rank test: *P* < 0.001), obesity (log-rank test: *P* = 0.005) and TNM stage (log-rank test: *P* < 0.001). The unadjusted HRs of the clinicopathological factors for OS are shown in Table 3.

**Table 3:** Association of LUNAR1 and clinical factors with overall survival in patients with CRC.

	Unadjusted HR* (95% CI)	*P*	Adjusted HR^†^ (95% CI)	*P*
LUNAR1 level	3.49 (1.98–6.14)	<0.001	2.79 (1.56–4.99)	0.001
Sex	0.98 (0.67–1.45)	0.942	0.89 (0.60–1.34)	0.584
Age	1.12 (0.76–1.64)	0.572	1.11 (0.75–1.65)	0.607
Tumour location	1.25 (0.82–1.73)	0.763	1.03 (0.65–1.69)	0.902
Tumour size	1.53 (0.98–2.39)	0.061	1.26 (0.59–2.71)	0.511
Differentiation status	2.13 (1.23–3.68)	0.007	1.16 (0.63–2.15)	0.626
TNM stage	3.19 (1.43–7.14)	<0.001	3.54 (1.54–8.14)	0.003

*Hazard ratios in univariate models.

^†^Hazard ratios in multivariable models.

Abbreviations: HR, hazard ratio; 95% CI, 95% confidence interval.

In multivariate analysis, increased LUNAR1 levels were characterized as an independent unfavourable prognostic factor in patients with CRC after adjusting for known prognostic factors. In CRC patients with increased LUNAR1 expression, the adjusted HR of unfavourable prognosis was 2.79 (95% CI: 1.56–4.99, *P* < 0.001) compared with that of patients with tumours of preserved LUNAR1 expression. The adjusted HRs of the clinicopathological factors for OS are shown in Table 3.

### Inhibition of LUNAR1 suppresses tumour cell proliferation and invasion

**Figure 4:**
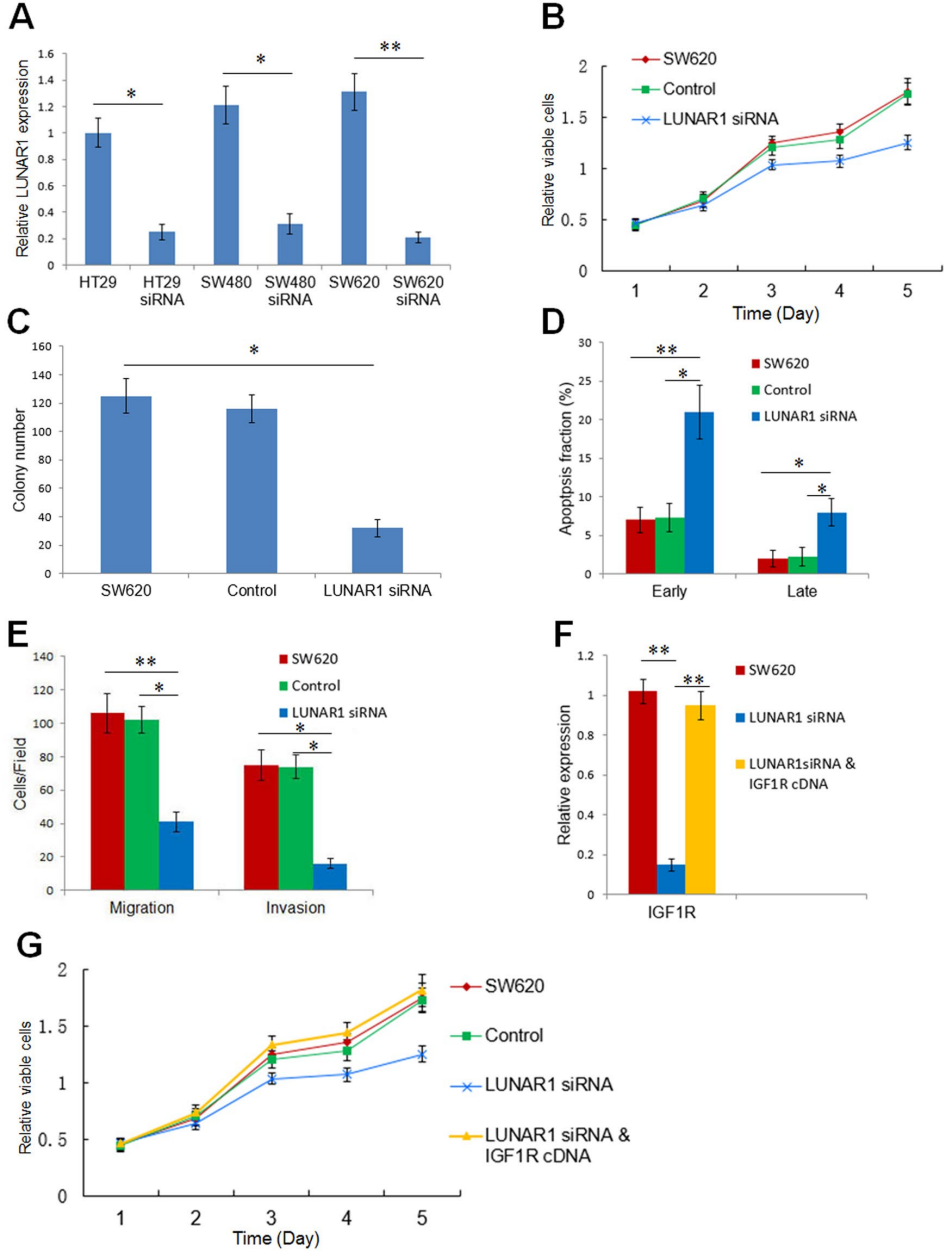
Inhibition of LUNAR1 suppresses the proliferation and invasion of colorectal cancer cells via IGF1 signalling. (**A**) Downregulation of LUNAR1 by siRNA verified by real-time PCR. The relative expression of LUNAR1 was analyzed using the 2^−ΔΔCt^ method. (**B**) The proliferation of cells detected by CCK-8 reagent. The data represent the results of three independent experiments. (**C**) Colony formation assay of three independent experiments. (**D**) The percentage of early apoptotic cells (Annexin V-positive and PI-negative) and late apoptotic cells (Annexin V-positive and PI-positive); the data represent the results of three independent experiments. (**E**) Migration and invasion assays; the counts of cells are presented as the mean values per field from at least five randomly-selected low-powered fields (×200) from three independent experiments (error bars: Means ± SDs). (**F**) IGF1R expression was decreased after LUNAR1 siRNA transfection, and increased after ectopically expressing vector containing IGF1R; (**G**) The proliferation of CRC cells detected by CCK-8 reagent indicated that ectopically IGF1R expression after LUNAR1 interference reversed the results of LUNAR1 siRNA transfection alone, the data represent the results of three independent experiments. (*P < 0.05, **P < 0.001).

After transfection of LUNAR1 siRNA, real time PCR confirmed that LUNAR1 expression was the highest in SW620 cells, and significantly decreased in the stably transfected cells compared with control cells (Fig. 4A). Therefore, we selected SW620 cells for further investigation. The proliferation of SW620 cells was significantly suppressed by the downregulation of LUNAR1, as measured by the CCK-8 reagent responses in the cell viability assay (Fig. 4B). To further investigate the impact of LUNAR1 on proliferation, colony formation and flow cytometry assays were utilized on LUNAR1 siRNA-transfected cells and control cells. The results showed that the number of cancer cell colonies was decreased in LUNAR1-downregulated cells, and LUNAR1 siRNA transfection caused a higher percentage of apoptotic cells (Fig. 4C,D). These results indicated that LUNAR1 could promote CRC cell growth by suppressing apoptosis. Because the association of LUNAR1 was found to be associated with tumour aggressiveness in clinical specimens, we next performed a transwell assay to confirm the effect of LUNAR1 on the migration and invasion of cells. The results showed that downregulation of LUNAR1 attenuated the capacity of migration and invasion in SW620 cells (Fig. 4E). To validate the enhancing effect of LUNAR1 on IGF1 signalling, we next evaluated the expression alteration of IGF1R after the downregulation of LUNAR1. The results showed that IGF1R was consequently decreased in LUNAR1-interfered SW620 cells (Fig. 4F). To further convince that LUNAR1 contributes to CRC progression through regulation of IGF1 signal in a rescue manner. We performed RNAi mediated depletion of LUNAR1 in CRC cells followed by ectopically expressing of IGF1R (Fig. 4F). Results on CRCs proliferation showed that ectopically IGF1R expression after LUNAR1 interference reversed the results of LUNAR1 siRNA transfection (Fig. 4G).

## Discussion

CRC is one of the most common malignant tumours worldwide. Even after surgical resection and adjuvant therapy, the prognosis of CRC is still unfavourable. Therefore, the accurate prediction of prognosis is necessary to better and more precisely manage CRC^13,14^. In addressing this challenge, our present study provided novel insight to explore new potential molecular markers for prognosis predication and as measures to evaluate the therapeutic effectiveness of medical interventions^15,16^.

The potential function and clinical utility of the Notch signal-induced long noncoding RNA LUNAR1 in CRC is the primary purpose of the present investigation. Considering the paradox of the complex function and simplicity of the target gene, we assume that this Notch signal-induced lncRNA might play significant roles in CRC. The investigation of LUNAR1 expression in clinical specimens indicated that its level in CRC was significantly increased compared with that in adjacent normal specimens. Moreover, the increased expression pattern of LUNAR1 was more likely to be detected in tumours with a poor differentiation status or an advanced TNM stage. These results suggested that LUNAR1 might act as an oncogene and play a significant role in cancer progression. Therefore, elucidating whether LUNAR1 expression in CRC could predict the DFS and OS of patients with CRC could further determine its potential clinical utility. Next, we conducted univariate and multivariate survival analyses to evaluate the prognostic role of LUNAR1 in CRC. The Kaplan-Meier analysis and Cox proportional hazards model analysis resultssuggested that increased LUNAR1 expression levels in CRC were independently associated with unfavourable DFS and OS outcomes after controlling for known prognostic factors. In this context, LUNAR1 might constitute an independent biomarker of tumour recurrence and prognosis for CRC patients and could contribute to precise diagnosis and treatment. As LUNAR1 is a Notch signal-induced lncRNA, our results also indicated that the function of LUNAR1 could be a part of Notch signalling. To explore the cellular mechanism of this Notch-induced lncRNA, we conducted an investigation using LUNAR1 interference in colorectal cell lines. The results showed that the proliferation, colony formation and invasion of colorectal cancer cells were significantly inhibited after the downregulation of LUNAR1, while cell apoptosis was induced. We also performed RNAi mediated depletion of LUNAR1 in CRC cells followed by ectopically expressing of IGF1R, in order to confirm the mechanism of LUNAR1 in a rescue manner. Results of CRC cells proliferation showed that ectopically IGF1R expression after LUNAR1 interference reversed the results of LUNAR1 siRNA transfection, indicating that LUNAR1 contributes to the CRC progression through regulation of IGF1 signal. These results further support the oncogenic role of LUNAR1 in colorectal cancer.

Recently, a series of investigations demonstrated that lncRNAs are aberrantly expressed in various human malignancies^17,18^. Given the complex roles of Notch signalling and its few known direct target genes, this lncRNA might be a potential functional target of the Notch pathway. We sought to determine whether LUNAR1, a Notch-induced lncRNA first reported in T-ALL, plays a significant role in colorectal cancer. Our investigation revealed that LUNAR1 siRNA transfection could inhibit IGF1R expression, confirming the sustaining effect of LUNAR1 on IGF1 signalling. Accumulating evidence has suggested that the over-activation of IGF1 signalling contributes to the development and progression of colorectal cancer^19^.

The present study, including clinical specimens and cell lines, provided the first piece of evidence suggesting that the increased LUNAR1 expression pattern in CRC may serve as an independent predictor of tumour recurrence and prognosis through its regulation of proliferation, indicating that the function of the lncRNA-mediated non-canonical pathway constitutes part of the Notch signal in CRC.

## Methods

### Clinical specimens

The protocols utilized in this study were approved by the ethics committees of Xi’an Jiaotong University and the Fourth Military Medical University. All patients recruited in this study provided informed consent. All research was performed in accordance with the relevant guidelines and regulations. The participants were randomly recruited from those who were consecutively diagnosed with CRC and underwent surgical resection between February 2010 and November 2012 at the Xijing Hospital of Digestive Diseases, the Fourth Military Medical University (Xi’an, China). Clinical CRC specimens were collected by surgeon in 10 min after surgical resection and put into liquid nitrogen for 10 min, then into a −80 °C ultra-freezer for mRNA isolation. The histology of these specimens had been double checked and confirmed by pathologists. The clinicopathological data and follow-up information of the participants were prospectively entered into a database, which was updated according to the survival status of participants by trained nurses every six months via telephone interview or questionnaire letters. Patients who had invalid census data, non-adenocarcinoma, inaccurate follow-up, missing data regarding pathological information or withdrew from the survey were excluded.

### Study endpoints

Disease-free survival (DFS) was defined as the time elapsed from surgery to the first occurrence of any of the following events: CRC distant metastasis, recurrence of CRC, or the development of a second non-colorectal malignancy excluding basal cell carcinomas of the skin and carcinoma *in situ* of the cervix; or death from any cause without documentation of a cancer-related event. The diagnosis of recurrence and distant metastasis was based on imaging methods such as ultrasonography, computed tomography, magnetic resonance imaging and position emission tomography and, if possible, cytologic analysis or biopsy. Overall survival (OS) was defined as the time elapsed from surgery to death of patients with CRC.

### Real-time PCR, mutation and MSI investigation

Total RNA from clinical CRC specimens and normal tissue specimens was purified using Trizol reagent (Invitrogen, Carlsbad, CA) according to the manufacturer’s instructions. cDNA synthesis was performed with 5 µg of total RNA per 20 μL using a cDNA reverse transcription kit (Fermentas, Lithuania). Real-time PCR was carried out on an ABI 7700 system (Applied Biosystems) using SYBR Green I (Invitrogen, Carlsbad, CA) as described before^20^. The primers for LUNAR1 were forward 5′-GGAGGCTGAGGCCGCCTGTT-3′ and reverse 5′-AGGCTGCAGGGGAACAGGTCTT-3′. The IGF1R primers were forward 5′-TACTCGGACGTCTGGTCCTT-3′ and reverse 5′-TGGGGTTATACTGCCAGCAC-3′. The internal control 18S rRNA primers were forward 5′-CGCCGCTAGAGGTGAAATTC-3′ and reverse 5′-TTGGCAAATGCTTTCGCTC-3′. The relative expression of target gene, normalized to 18S rRNA, was analyzed using the 2^−ΔΔCt^ method. DNA extraction, microsatellite instability (MSI) analysis, and the pyrosequencing of KRAS, BRAF and PIK3CA were performed as described before^21–23^.

### Cell Lines and transfection

Colorectal cancer cell lines utilized in the present study are HT29, SW480 and SW620, which was purchased from the Chinese Academy of Sciences Cell Bank (Shanghai, China). According to the instructions for these cell lines, the cells were cultured in L-15 or 1640 medium (Invitrogen, Carlsbad, CA) supplemented with 10% foetal calf serum. To downregulate LUNAR1 expression in cells, LUNAR1 targeted stealth RNAi (LUNAR1 siRNA) was utilized (Invitrogen, Carlsbad, USA). The sequence of the LUNAR1-targeted small interfering RNA was GATTCTGTAGCCTAACAAGCTTTCCAGTGATT. During transfection, according to the manufacturer’s instructions, the cells were transfected with 50 nmol of LUNAR1 siRNA using Lipofectamine 2000 transfection reagent (Invitrogen, Carlsbad, USA) with a scrambled negative control. After transfection of LUNAR1 siRNA, cells were also transfected with plasmids containing IGF-1R (pcDNA3.1-IGF1R) and empty vector (GenePharma, Shanghai, China). The transfected cells were harvested after 48 h of incubation for real-time PCR investigation to evaluate the transfection efficiency and target gene expression.

### Cell viability, colony formation and apoptosis assays

The Cell Counting Kit-8 (Dojindo, Kumamoto, Japan) was utilized to evaluate cell proliferation by counting viable cells. After screening, LUNAR1 siRNA stably transfected cells and controls were plated into 96-well plates. During an incubation of 5 consecutive days, the culture medium for both stably transfected cells and controls was changed and replenished with 10 μl of CCK-8 reagent and 90 μl of fresh L-15 medium. Then, after incubation at 37 °C for 1 h, the 96-well plates were evaluated by a microplate reader (Pharmacia Biotech) according to the manufacturer’s instructions. In addition, in the colony formation assay, LUNAR1 siRNA stably transfected cells and controls were plated into 6-cm plates (200 cells per plate) and cultured in L-15 medium (Invitrogen, Carlsbad, CA) with 10% foetal calf serum. After 2 weeks, the colonies in the plates were fixed with cold methanol and stained with 1% crystal violet. The FITC Annexin V Apoptosis Detection Kit (BD Pharmingen, USA) was utilized to evaluate the difference in apoptosis between LUNAR1 siRNA-transfected cells and controls by flow cytometry. LUNAR1 siRNA-transfected cells and controls were harvested every 12 h within 48 h after transient transfection and washed in PBS and then stained with Annexin V and propidium iodide. The percentage of apoptotic cells in the LUNAR1 siRNA-transfected cells and controls was quantified by a BD FACS Verse flow cytometer. Each experiment above was repeated in triplicate.

### Migration and invasion assays

Transwell experiments were utilized for the evaluation of migration and invasion. Specifically, 8-μm transwell polycarbonate insert chambers (BD Biosciences, San Jose, CA) coated with and without 40 μl of Matrigel (Corning, NY, CA) were prepared. Then, the chambers were incubated at 37 °C for 2 h to solidify the Matrigel. Afterwards, the same density (1 × 10^5^ in 100 μl) of LUNAR1 siRNA-transfected cells and control cells in either serum-free DMEM/F12 or DMEM-high glucose were added into the upper compartment of the transwell polycarbonate insert chambers. In the bottom chambers, 600 μl of conditioned medium for cell use was placed as a chemoattractant. The medium in the upper chamber was removed after incubation at 37 °C and 5% CO_2_ for 24 h. The invaded LUNAR1 siRNA-transfected cells and control cells in the lower membrane were fixed with 4% paraformaldehyde and stained with 0.1% crystal violet (Invitrogen, Carlsbad, CA).

### Statistical analysis

SPSS (version l6.0) software was utilized for statistical analysis. The associations of LUNAR1 levels with clinicopathological characteristics were investigated by the Pearson *χ*^2^ test or Fisher’s exact test. Kaplan-Meier univariate survival analysis was utilized to identify potential prognostic factors for the survival of CRC patients. In the multivariate Cox proportional hazards model analysis, independent prognostic factors were justified by adjusting for known prognostic factors. Statistically significant differences were defined as a *P* value of 0.05 or less.
